# Fluorescence immunosensor based on functional nanomaterials and its application in tumor biomarker detection

**DOI:** 10.1039/d2ra04989a

**Published:** 2022-11-01

**Authors:** Juanjuan Huang, Fenghuang Wei, Yuling Cui, Li Hou, Tianran Lin

**Affiliations:** School of Chemistry and Pharmaceutical Science, State Key Laboratory for the Chemistry and Molecular Engineering of Medicinal Resources, Guangxi Normal University Guilin 541004 P. R. China houli@gxnu.edu.cn tianranlin@163.com; Jinan Center for Food and Drug Control Jinan 250102 Shandong China

## Abstract

An immunosensor is defined as an analytical device that detects the binding of an antigen to its specific antibody by coupling an immunochemical reaction to the surface of a device called a transducer. Fluorescence immunosensing is one of the most promising immunoassays at present, and has the advantages of simple operation, fast response and high stability. A traditional fluorescence immunosensor often uses an enzyme-labelled antibody as a recognition unit and an organic dye as a fluorescence probe, so it is easily affected by environmental factors with low sensitivity. Nanomaterials have unique photostability, catalytic properties and biocompatibility, which open up a new path for the construction of stable and sensitive fluorescence immunosensors. This paper briefly introduces different kinds of immunosensors and the role of nanomaterials in the construction of immunosensors. The significance of fluorescent immunosensors constructed from functional nanomaterials to detect tumor biomarkers was analyzed, and the strategies to further improve the performance of fluorescent immunosensors and their future development trend were summarized.

## Abstract 2

Fluorescence immunosensors constructed from functional nanomaterials for the detection of tumor biomarkers such as enzymes, RNA, DNA, proteins and cancer cells were summarized.

## Graphical abstract

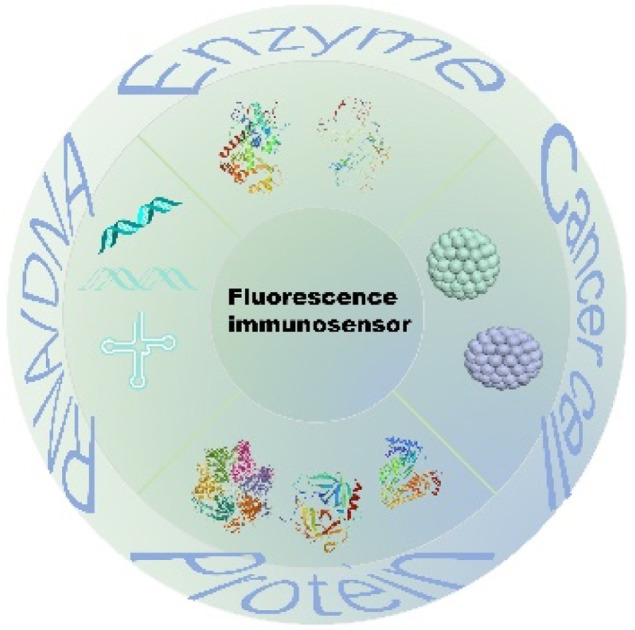

## Immunosensor

### Brief introduction of immunosensors

In recent years, as an effective detection tool, immunosensors have been widely used in medical diagnosis, food safety analysis, environmental monitoring and other fields.^1,2^ An immunosensor is defined as an analytical device that detects the binding of an antigen to its specific antibody by coupling an immunochemical reaction to the surface of a device called a transducer.^3^ The biological elements of general immunosensors are composed of antibodies or antigens. Through the immune complex formed by the combination of an antigen and antibody, the sensing platform is constructed for signal transduction.^4^ The highly specific recognition between antigen and antibody makes the immunosensor have good stability and sensitivity, and can be used to detect low abundance biomolecules, such as bacteria, viruses, tumor biomarkers, nucleic acids and some other small molecules.^5,6^ At present, the immunosensor has the advantages of high performance-to-price ratio, less reagents and samples, simple operation and high sensitivity,^7^ which shows a broad development prospect in medical diagnosis. Specific protein molecules called tumor biomarkers are detected in the serum and body tissues of cancer patients.^8^ Tumor biomarkers mainly exist in tumor tissue or serum, and tumor and normal tissue can be distinguished by measuring tumor biomarkers in blood or secretion.^9^ Because of the sensitivity and specificity of immunosensor, tumor biomarkers can be used as a research object for sensitive detection and staging of solid tumors.^10,11^

### Classification of immunosensor

The classification method of immunosensor can be divided into two kinds of methods. Firstly, according to whether the antigen and antibody are labelled or not, it can be divided into labelled immunosensor and unlabeled immunosensor.^12^ Secondly, according to the different signals generated by immunosensor, it can be divided into optical immunosensor and electrochemical immunosensor.^13^ The optical immunosensor also includes chemiluminescence (CL), electrochemiluminescence (ECL), fluorescence (FL), colorimetry (CM), surface plasmon resonance (SPR) and surface enhanced Raman scattering (SERS).^14^ Then, colorimetric, electrochemical, electrochemiluminescence and fluorescence immunosensors are m ainly introduced below.

#### Colorimetric immunosensor

Colorimetry is a method to detect analytes by detecting the color changes of the solution, and the results can be quantitatively analyzed by spectroscopy.^15^ Colorimetric immunosensor is mainly composed of antibody recognition element and colorimetric probe, which does not need large and complex instruments in the detection process, and has the characteristics of simple operation and visualization.

**Fig. 1:**
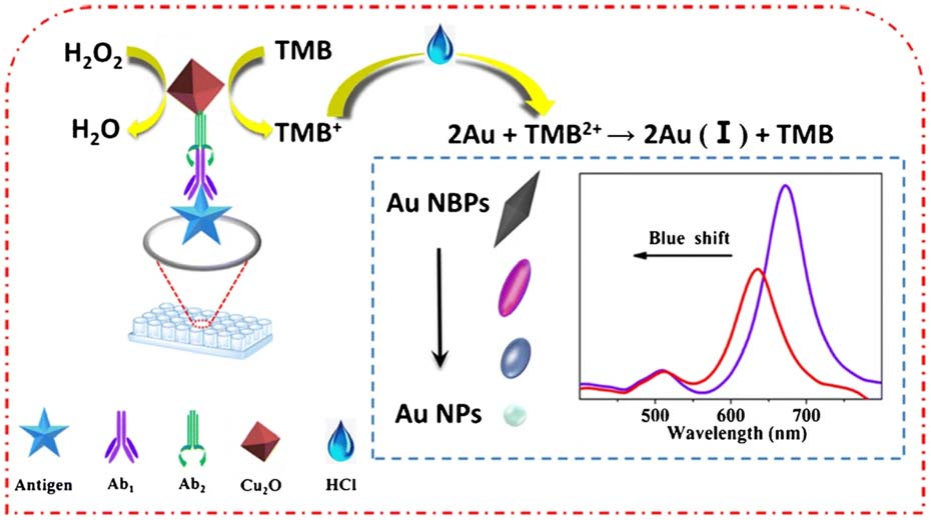
Schematic diagram of polychromatic immunosensor for OTA detection. Reproduced from ref. 21 with permission from Springer Nature.

In colorimetric immunoassay, enzyme-catalyzed chromogenic substrate is generally used to detect analytes, among which horseradish peroxidase (HRP) and alkaline phosphatase (ALP) are the two most commonly used enzymes.^16^ However, the above two enzymes are natural enzymes, which are expensive and easy to be inactivated in actual detection. Thus, the application of colorimetric immunosensor in complex detection environment is limited. Nanozyme, a nanomaterial with enzyme-like activity, has been widely used because of its high stability, good biocompatibility, mass preparation, convenient storage, low cost and easy modification.^17,18^ The emergence of nano-enzyme overcomes the defects of natural enzyme in the sensor,^19^ and it still maintains strong stability in the harsh environment. Therefore, nanozyme can replace natural enzyme to construct colorimetric immunosensor for the detection of biomolecules. Gold nanoparticles (AuNPs) is a kind of nanozyme with good performance, but the enzyme activity of AuNPs will be affected when a specific biometric element is immobilized. Therefore, many composite nanomaterials used to improve the enzyme-like activity of AuNPs were studied. For example, Fang *et al.*,^20^ used bio-functional M13 bacteriophage as a nanozyme carrier to load a large number of silver-coated gold nanoparticles (AuNPs@Ag) for sensitively colorimetric detection of deoxynivalenol (DON). The above methods are monochromatic detection, the same color change is not obvious, it will be difficult to carry out the naked eye semi-quantitative determination. Therefore, it is necessary to develop a sensitive polychromatic immunoassay. As shown in Fig. 1, Zhu *et al.*,^21^ established a polychromatic immunosensor based on gold nano-bipyramids (Au NBPs) mimetic enzyme etching for the detection of ochratoxin A (OTA). The 3,3′,5,5′-tetramethylbenzidine (TMB) was oxidized to form TMB^+^ by octahedral cuprous oxide (Cu_2_O). Then, the TMB^+^ was oxidized to TMB^2+^ by hydrochloric acid (HCl) and the Au NBPs was etched. The local surface plasmon resonance of etched Au NBPs resulted in an obvious blue shift, which made the polychromatic change of Au NBPs mixture. Although colorimetric detection is simple and rapid, there are still some problems that need to be improved, such as unstable colorimetric reagents, easy to be disturbed by colored matrix, low accuracy and so on.

#### Electrochemical immunosensor

**Fig. 2:**
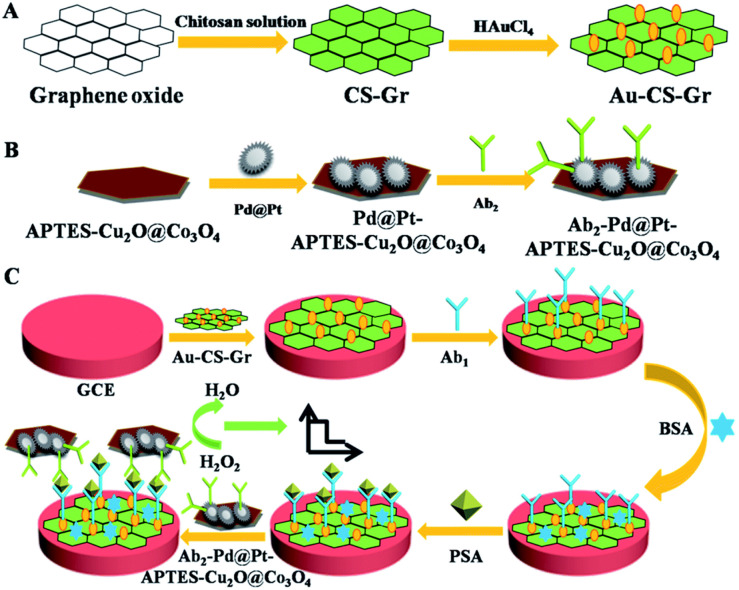
Schematic diagram of the principle of electrochemical immunosensor for PSA detection based on multi-signal amplification. Reproduced from ref. 25 with permission from Royal Society of Chemistry.

Electrochemical technology has good precision, accuracy and sensitivity, and can detect trace samples. It is a powerful and widely used tool. Electrochemical immunosensor is a kind of biosensor which combines electrochemical technology and immunoassay technology. It causes changes in potential, current, impedance, conductance, capacitance and ion concentration through immune reaction. Thus, the signal can be captured from the changes of current, conductance or impedance to achieve the purpose of electrochemical detection. The main analytical methods of electrochemical immunosensor are conductance method, potential method, current method and electrochemical impedance method.^22^ Electrochemical immunosensor has the advantages of rich signal changes, simple, convenient and high sensitivity. Therefore, it shows great potential in the field of food analysis and medical diagnosis. In recent years, a variety of electrochemical immunosensors have been successfully used for the detection of various biomolecules. Su *et al.*,^23^ designed a label-free electrochemical immunosensor based on gold nanoparticles/thionine/molybdenum disulfide (AuNP-Thi-MoS_2_) nanocomposites for the detection of carcino-embryonic antigen (CEA). In this method, the AuNP-Thi-MoS_2_ material was modified on the electrode surface, then the antibody was immobilized, and then the reaction between antigen and antibody reduced the current signal of Thi on the material. The step is simple and fast. In view of the development of nanomaterials, labeled nanomaterials can be effectively used for electrochemical signal amplification. Zhao *et al.*,^24^ designed a polymer brush/graphene oxide composite labeled secondary antibody. Through the construction of a sandwich electrochemical immunosensor, many signal labels on the P(VT-*co*-HEMA)-*g*-GO successfully amplified electrochemical signals and realized the sensitive detection of alpha fetoprotein (AFP). Based on the use of many different types of nanomaterials for secondary antibody labeling, Liu *et al.*,^25^ developed an electrochemical immunosensor with multiple signal amplification. In this work, the authors prepared 3-aminopropyl-triethoxysilane functionalized cuprous oxide@cobalt oxide nanocomposites immobilized fluffy spherical palladium@platinum nanoparticles (Pd@Pt-APTES-Cu_2_O@Co_3_O_4_) and used as multiple signal amplification label. As shown in Fig. 2. Cu_2_O@Co_3_O_4_ nanocomposites have high electron transfer ability and electrocatalytic performance, Pd@Pt has high electrocatalytic activity for H_2_O_2_ reduction, and the antibody and Pt can form a stable Pt–N covalent bond. The synthesized Pd@Pt-APTES-Cu_2_O@Co_3_O_4_ can improve the catalytic activity and electron transfer efficiency through the synergistic action of each component. This multiple signal amplification performance significantly improves the stability and sensitivity of the electrochemical immunosensor, and shows a wide linear range and low detection limit for the detection of prostate specific antigen (PSA). Although electrochemical immunosensor has made good progress in the detection and analysis of tumor markers, the electrode often needs to be modified for the construction of electrochemical immunosensor. The modified material on the electrode surface is easy to fall off in the detection process, resulting in the poor of the repeatability of the sensor, which limits the application of electrochemical immunosensor to a certain extent.

**Fig. 3:**
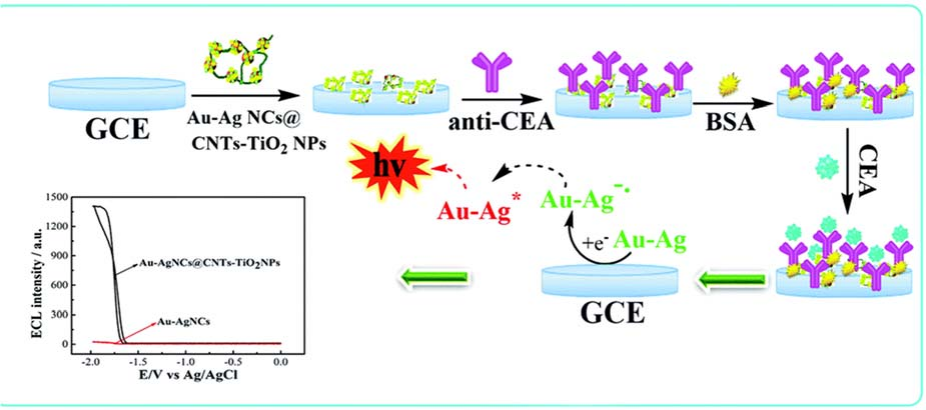
Schematic diagram of ultra-sensitive detection of CEA by immunosensor. Reproduced from ref. 33 with permission from Royal Society of Chemistry.

Electrochemiluminescence immunosensor In electrogenerated chemiluminescence, also known as electrochemiluminescence (ECL), electrochemically generated intermediates undergo a highly exergonic reaction to produce an electronically excited state that then emits light.^26^ Combining the advantages of electrochemical and spectral methods, the ECL has the following advantages: first, ECL does not need a light source and cannot be affected by light scattering and luminous impurities; second, the light emitter can be regenerated *in situ*, and the reaction between it and excessive co-reactants can prevent problems related to side reactions;^27^ third, the measurement time is short, only a few seconds; fourth, the reaction time and electrode surface space can be controlled. Fifth, the potential can be applied alternately to modulate the excited state.^28^ Therefore, the ECL immunosensor has high sensitivity, high specificity and good reproducibility, and is very competitive in the detection of analytes. ECL technology is mainly affected by three factors, namely, luminescence, co-reactants and electrodes. The development of new ECL luminescence has always been the focus of research. Because of the high ECL efficiency, ruthenium(ii) complex (Ru (bpy)_3_^2+^) and Luminol are two classical ECL phosphors.^29,30^ In recent years, nanomaterials have developed rapidly and have been successfully used as carriers of ECL light emitters, which effectively improve the properties of ECL. For example, Chang *et al.*,^31^ designed a novel dual-sensitization ECL immunosensor. In this method, Ru(bpy)_3_^2+^-doped chitosan/SiO_2_ nanoparticles were used as the first signal enhancers, and chitosan modified perfluoro sulfonic acid/multiwall carbon nanotubes (Nafion/MWNTs) composites were used as the second sensitizing matrix to seize large amounts of prostate specific capture antibody, which significantly enhanced the ECL reaction efficiency produced by Ru(bpy)_3_^2+^. The highly sensitive determination of PSA has been realized. In addition, some nanomaterials have become excellent ECL phosphors because of their excellent biocompatibility and stability, such as quantum dots, metal nanoclusters and carbon nanomaterials.^32^ However, quantum dots contain heavy metals such as lead or cadmium, which are toxic and dangerous in biological applications. Although the biocompatibility of metal nanoclusters is good, their ECL efficiency is relatively low. Therefore, Huang *et al.*,^33^ constructed an ECL immunosensor for the ultrasensitive detection of CEA using gold–silver bimetallic nanoclusters/carbon nanotube/titanium dioxide nanocomposites (Au–AgNCs@CNTsTiO_2_NPs). The detection mechanism is shown in Fig. 3. In Au–AgNCs@CNTsTiO_2_NPs nanomaterials, TiO_2_ acted as a co-reaction promoter and CNTs acted as an electronic conductor, which improved the electron transfer rate of the immunosensor and achieved more effective energy transfer, thus obtaining a more significant ECL effect. This method is non-toxic, stable and has good ECL effect.

Although the ECL immunosensor is sensitive, stable and reproducible, the detection process of ECL is relatively complex, and most of them need to add H_2_O_2_. In addition, the damage caused by H_2_O_2_ to biomolecules and other stability factors will affect the sensitivity and stability of the sensor. Therefore, it is very important to construct a H_2_O_2_-free ECL immunosensor.

#### Fluorescence immunosensor

**Fig. 4:**
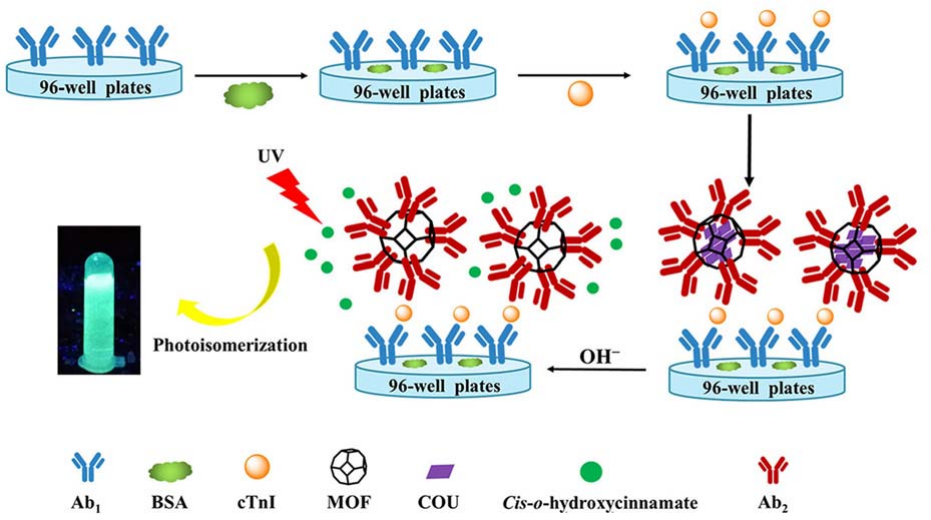
Schematic diagram of cTnI detection by fluorescence immunosensor with signal amplification. Reproduced from ref. 38 with permission from American Chemical Society.

Fluorescent immunosensor, based on immune reagents as molecular recognition units, fluorescent reagents or enzymes as labels, which achieves the determination of antigen or antibody through the specific reaction between antibody and antigen. Because of its simple operation, fast response and high stability in practical detection, fluorescence immunosensor analysis is one of the most promising immunoassays at present.^34^ Organic dyes are often used as fluorescent probes for fluorescence immunosensors, such as calcitonin^35^ and fluorescein isothiocyanate (FITC) due to their low cost and easy to obtain.^36^ However, these organic fluorescent dyes have some defects, such as hydrophobicity, easy photobleaching degradation, short fluorescence lifetime, low sensitivity and so on. In order to overcome these shortcomings, researchers have made great contributions to the improvement of fluorescent probe performance. Zhang *et al.*,^37^ designed a fluorescence immunosensor based on bifunctional DNA nano-machine to detect circulating tumor cells. Carboxyfluorescein (FAM), fluorescence quenching agent and hexachlorofluorescein (HEX) were used to modify the substrate probes and assistant probes, respectively, and the dendritic DNA product with a large number of FAM molecules was used to image the target cells by binding to the aptamer. The sensitive detection of circulating tumor cells was realized. However, fluorescein-labeled DNA is expensive and complex to design. Therefore, Wang *et al.*,^38^ designed a coumarin encapsulated metal–organic framework (MOF@COU) to build a fluorescence signal amplification immunosensor platform to detect cardiac troponin I (cTnl). As shown in Fig. 4, MOF@COU was fixed to a 96-well plate by double antibodies, and COU was hydrolyzed to form hydrophilic *cis-o*-hydroxycinnamate under alkaline conditions and released from MOF. The hydrolysate was photoisomerized to form fluorescent *trans-o*-hydroxycinnamate under UV excitation. MOF encapsulated a large number of COU to achieve the function of signal amplification, and the hydrophobicity of the metal–organic frame helped to retain the performance of COU, ensuring the stability and repeatability of the immunosensor, and realizing the sensitive detection of cTnl.

**Fig. 5:**
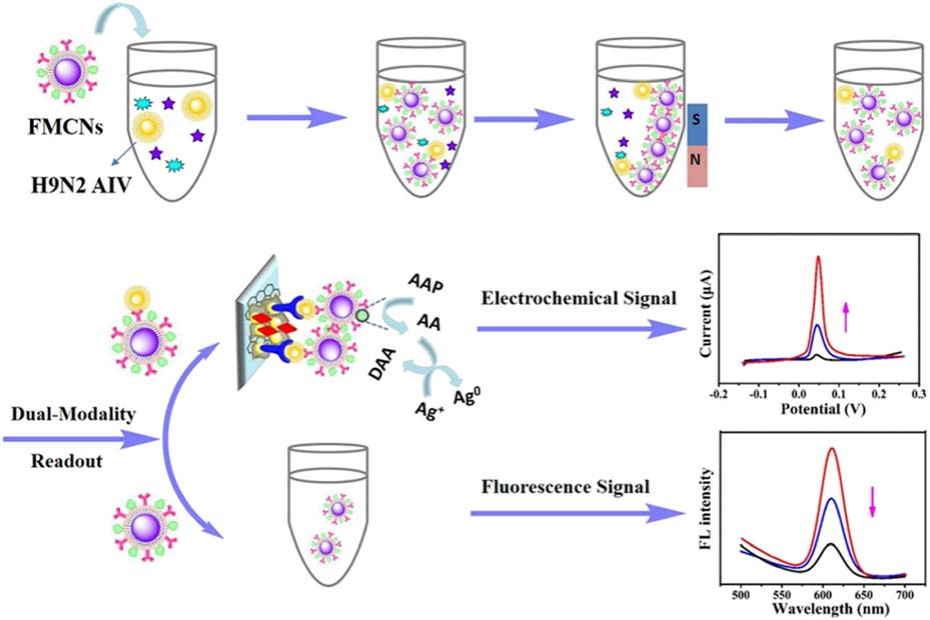
Schematic diagram of fluorescence and electrochemical dual-mode immunoassay platform for detection of avian influenza virus. Reproduced from ref. 39 with permission from American Chemical Society.

In recent years, in addition to being used as carriers for fluorescent probes, some nanomaterials with unique light stability, biocompatibility and strong fluorescence under ultraviolet lamp have been successfully used as fluorescence probes for immunosensors, such as quantum dots,^39^ metal nanoclusters,^40^ upconversion nanoparticles^41^ and so on. These nanomaterials have a variety of physical and chemical properties, and nanocomposites can be synthesized and designed to further improve their properties. For example, carbon quantum dots (CQDs) can enhance the fluorescence of CQDs/ZnO nanocomposites by modifying zinc oxide (ZnO).^42^ With the extensive exploration of these nanocomposites, multi-functional nanomaterials can be combined with other immunosensor. As shown in Fig. 5, Peng *et al.*,^43^ designed a fluorescent and electrochemical dual-mode immunoassay platform based on fluorescent-magnetic-catalytic nanospheres (FMCNs) to detect H9N2 avian influenza virus (H9N2 AIV). The multifunctional nanomaterial FMCNs was prepared by assembling ferric oxide nanoparticles and quantum dots on the surface of the copolymer nanospheres. The surface of the material has rich carboxyl groups and large specific surface area, which can modify ALP and monoclonal antibody molecules at the same time. Based on the specific reaction of antigen and antibody, the target can be captured from complex samples to form the complex of target and FMCNs, which can be separated by magnetic separation. The complex and excess FMCN were separated and enriched rapidly. After separation, the complex was captured by the ITO electrode labeled with polyclonal antibody, and the ALP on FMCNs catalyzed the silver deposition reaction to obtain the electrochemical signal. The supernatant of FMCNs without binding to the target was separated and used for fluorescence signal detection. This method has high accuracy, diversity and flexibility, and can meet the detection needs of different regions and different situations. Therefore, the fluorescent immunosensor is simple, sensitive, stable and has high applicability.

According to the different output signals, colorimetric immunosensor, electrochemical immunosensor, electrochemiluminescence immunosensor and fluorescence immunosensor are introduced respectively. For the construction of immunosensor, the design of sensing probe has a great influence on its analytical performance. As can be seen from the above introduction, with its excellent performance, nanomaterials are of great help to improve the stability, sensitivity, repeatability and accuracy of the immunosensor, and play an important role in the construction of the immunosensor.

## The role of nanomaterials in immunosensor

There are two main points in the construction of an immunosensor with good performance, the first is the fixation of biomolecules, and the second is the well-designed sensing probe. Due to their good biocompatibility, large specific surface area and excellent stability, nanomaterials can be used to effectively improve the immobilization of biomolecules on immunosensor.^44^ Using the unique optical properties or enzyme-like activity of some nanomaterials, they can be used as sensing probes to improve the stability and sensitivity of the sensing platform, and the signal amplification of the immune sensing platform can be realized through the design of some nanocomposites.^45^ With the introduction of nanomaterials, the design of immunosensor becomes more plentiful and flexible. The role of nanomaterials in immunosensors will be discussed below.

### Nanozyme

**Fig. 6:**
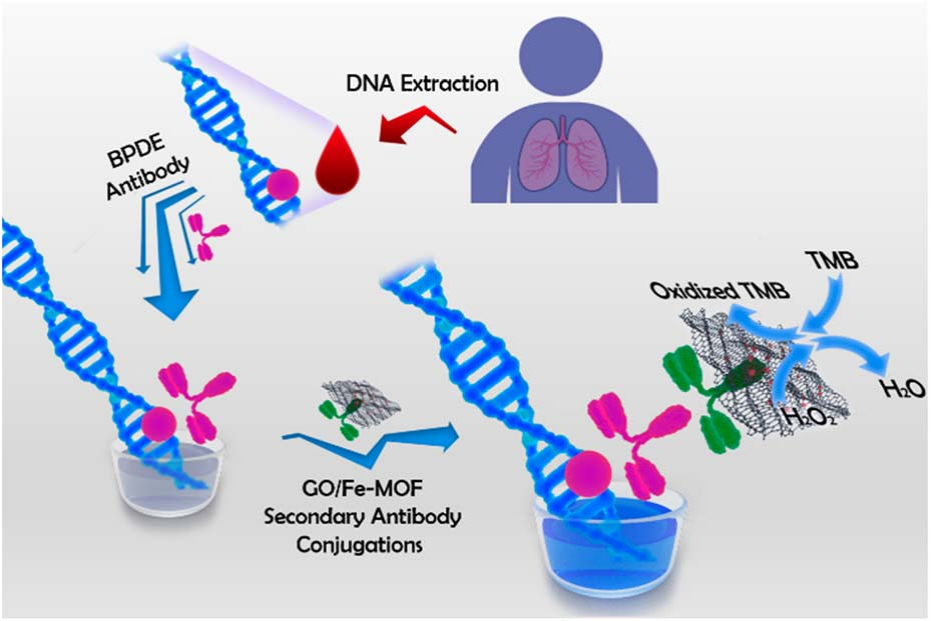
A colorimetric immunosensor constructed by nano-enzyme for ultrasensitive detection of BPDE–DNA in human blood. Reproduced from ref. 49 with permission from American Chemical Society.

At present, various nanomaterials with enzyme-like activity have been developed. Such as noble metal nanoparticles,^46^ metal oxides,^47^ magnetic nanoparticles,^48^ graphene oxide^49^ and metal organic framework materials^50^ and so on. Although monometallic nanozyme has enzyme-like catalytic activity, its catalytic activity is still not high enough, which leads to its low sensitivity for immunosensor detection. In order to improve the catalytic activity of these nanomaterials, a large number of polymetallic nanoparticles have been developed to improve this problem.^51^ For example, Liu *et al.*,^52^ prepared gold nanobipyramid coated Pt (AuBP@Pt) modified electrode and enhanced the electroactive area. Using AuPd alloy modified polydopamine (AuPd-PDA) to catalyze signal amplification, an ultrasensitive electrochemical immunosensor was constructed for the detection of human apolipoprotein E4. In addition, besides good catalytic activity, good dispersibility and water solubility are also necessary conditions for sensitive detection of biomolecules by nanozymes. As shown in Fig. 6, Ruan *et al.*,^53^ designed a nanozyme consisting of iron-based metal–organic framework (Fe-MOF) and graphene oxide (GO) for colorimetric analysis. In this study, positively charged Fe-MOF can be evenly distributed on the surface of negatively charged GO slices through electrostatic interaction. Because GO has good water solubility and biocompatibility, it greatly improves the water solubility of Fe-MOF, and the synthesized nanozyme can be well used for antibody modification. Because of the large surface area and abundant active sites of Fe-MOF and GO, the synthesized nanozyme has excellent peroxidase-like activity. Therefore, the colorimetric immunosensor has the function of double catalytic signal amplification. It provides a new idea for the development of ultra-sensitive immunosensor.

### Carrier of biomolecules

**Fig. 7:**
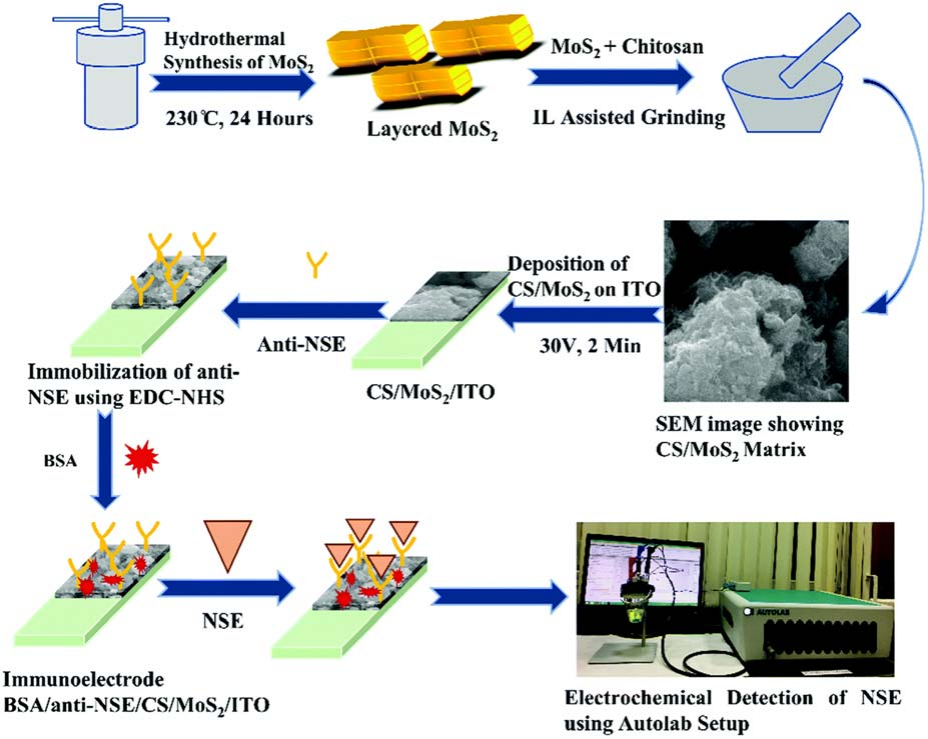
Schematic diagram of electrochemical immunosensing platform for NSE detection based on CS/MoS_2_. Reproduced from ref. 55 with permission from Royal Society of Chemistry.

The immobilization of biomolecules is a key step in the design of immunosensor. At present, the large specific surface area of nanomaterials provides favorable conditions for better adsorption of biomolecules. In immunosensor, the first condition for biomolecule immobilization is that nanomaterials have good biocompatibility and do not affect the inherent structure and function of biomolecules.^54^ Therefore, a large number of nanomaterials for biomolecule immobilization have been studied in recent years.^55–57^ The unique properties of AuNPs provide a multifunctional platform for biomolecules. Cheng *et al.*,^58^ designed an AuNPs-assisted enzyme signal amplification method, which was used in paper enzyme-linked immunosorbent assay (ELISA) to detect PSA in human serum. In view of the high affinity between polyadenine and AuNPs, a large number of biotinylated polyadenine ssDNA sequences were immobilized on the surface of AuNPs. Then the high density combination of biotin and horseradish peroxidase modified streptavidin was used to amplify the enzyme-catalyzed signal. The antibody was adsorbed on the surface of AuNPs to bind to the target, and the highly sensitive detection of PSA was successfully achieved. There are many nanomaterials commonly used as the matrix for the immobilization of antibodies on the immunosensor platform. As shown in Fig. 7, R. Khatri *et al.*,^59^ prepared chitosan-functionalized molybdenum disulfide nanomaterials (CS/MoS_2_) as the sensing matrix for fixing neuron-specific enolase (NSE) antibodies. Electrochemical immunosensor analysis of NES was carried out by simple antibody immobilization on CS/MoS_2_ and antigen–antibody specific recognition. The CS/MoS_2_ is a highly stable matrix, which makes the immobilized antibody maintain good biological activity and can be detected repeatedly, and the layered structure of MoS_2_ can increase the surface area of the immobilized antibody, thus realizing the repeatable and renewable electrochemical detection of NES.

**Fig. 8:**
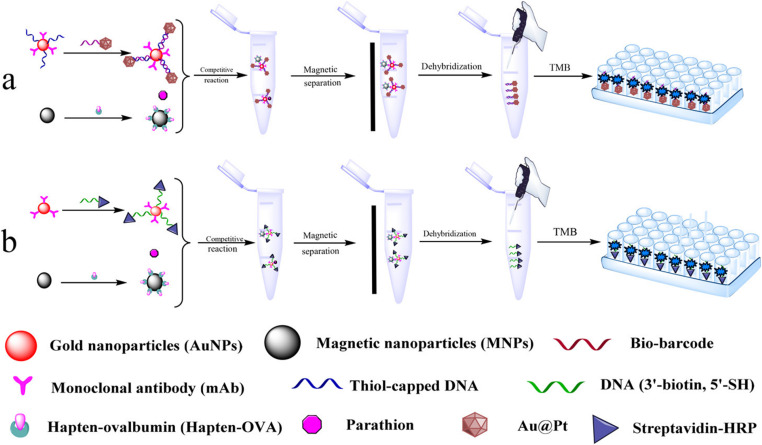
Schematic presentation of the colorimetric Bio-Barcode Immunoassays using (a) Au@Pt and (b) HRP amplification. Reproduced from ref. 60 with permission from American Chemical Society.

In addition, magnetic nanoparticles have been widely used to immobilize biomolecules and capture biomolecule modified nanoparticles, and magnetic nanoparticles can be used for more functional design. As shown in Fig. 8, Chen *et al.*,^60^ designed a competitive and sensitive bio-barcode immunoassay for parathion based on bimetallic nanozyme (Au@Pt). The construction of the sensing platform is mainly divided into two parts. On the one hand, the Au@Pt probes functionalized with the complementary thiolated ssDNA reacted with the Au NP probes that were modified with single-stranded thiol oligonucleotides and monoclonal antibodies to form nanozyme probes through complementary base pairing. On the other hand, ovalbumin–parathion hapten was coupled to magnetic nanoparticles (MNP) as MNP probes. Parathion competed with the MNP probe to bind the antibody on the nanozyme probe, supplemented by magnetic separation, so that the nanozyme probe bound to parathion can be used to catalyze the chromogenic system. In order to verify the feasibility of this method, a competitive sensitive bio-barcode immunoassay for parathion based on HRP was designed, and the sensitivity and stability of the two schemes were compared. In this sensing strategy, the introduction of magnetic nanomaterials can not only efficiently immobilize biomolecules, but also capture biomolecule-modified nanozymes and separate complex sample matrixes, which greatly promotes the stability of the immunosensor platform.

### Signal indicator

**Fig. 9:**
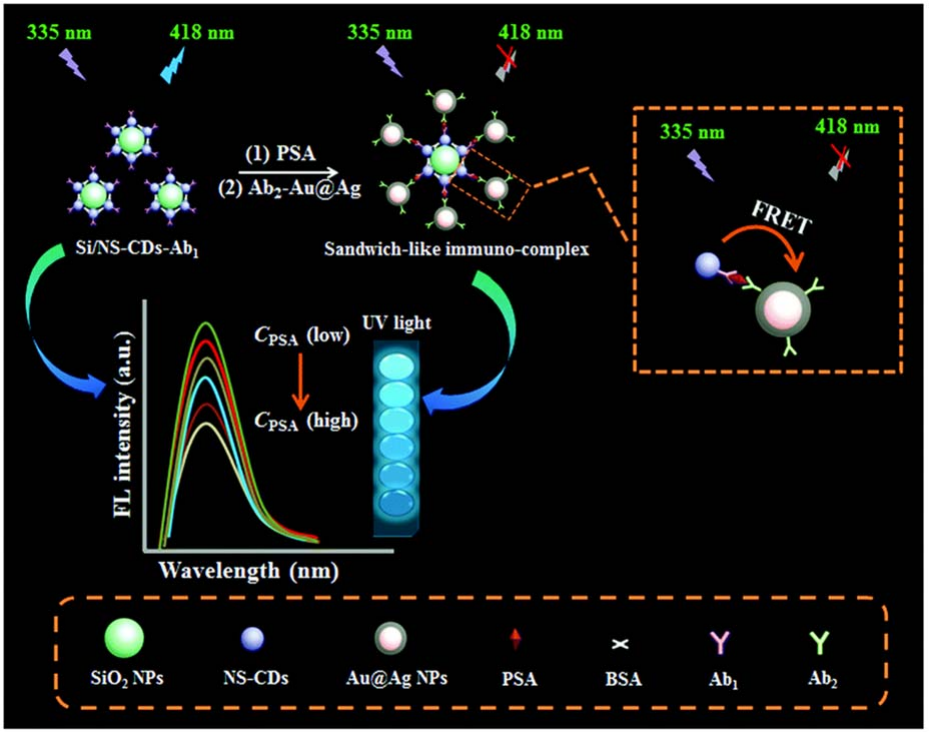
Schematic diagram of a novel fluorescence immunosensor for PSA sensitive detection based on resonance energy transfer. Reproduced from ref. 63 with permission from Royal Society of Chemistry.

Immunoassay is usually carried out in the form of competition or sandwiches. Traditional immunoassays often use natural enzymes, organic dyes, redox molecules, electroactive molecules and metal ions as signaling agents. The output signal is obtained by labeling antibodies or signaling agents.^54,61^ However, natural enzymes and organic dyes have some problems, such as poor stability in complex environment, high cost of sensor design, and low electron transfer efficiency of redox molecules, which limit their application in immunosensor. Because of the advantages of simple preparation, low cost and many types of signal output, nanomaterials can be used as an excellent signal indicator to couple with antibodies or antigens to form signal probes, which opens up a new way for the construction of immunosensors.^62^ As shown in Fig. 9, Cao *et al.*,^63^ constructed a novel fluorescent immunosensor for sensitive detection of PSA based on Förster resonance energy transfer between nitrogen and sulfur co-doped carbon quantum dots functionalized silica nanospheres (Si/NS-CDs) and Au@AgNPs. Si/NS-CDs had excellent fluorescence activity and low toxicity, so the first antibody labeled Si/NS-CDs was used as the fluorescence energy donor. The Au@AgNPs labeled with the second antibody was used as the fluorescent energy receptor. The sandwich immune binding of donor and recipient by adding antigen led to the closer distance between Si/NS-CDs and Au@AgNPs and the fluorescence resonance energy transfer. Therefore, the fluorescence of Si/NS-CDs decreased with the increase of PSA concentration.

In addition, there are nanomaterials as signal indicators in the field of colorimetry,^64^ chemiluminescence,^65^ electrochemistry,^66^ electrochemiluminescence^67^ and so on. For example, Wang *et al.*,^68^ used lanthanide (Ln) metal–organic frameworks (LMOFs) as a signal probe to construct a self-luminous ECL immunosensor for the detection of cytokeratin 21-1. LMOFs can be self-luminous and the emission energy is not disturbed with high electroluminescence efficiency.

## Fluorescence immunosensor based on functional nanomaterials for detection of tumor biomarkers

### The purpose and significance of detection of tumor biomarkers

Cancer is a complex disease characterized by unlimited proliferation and spreads of abnormal cells and is difficult to cure. It has always been the leading cause of death in many countries, causing about 9.6 million deaths in 2018.^69,70^ Therefore, cancer has caused great harm to human life and health. Cancer patients not only have to bear the pain caused by the disease, but also face a heavy economic burden. There is no doubt that timely and accurate detection is very important for clinical diagnosis, monitoring and prognostic treatment of cancer.

**Fig. 10:**
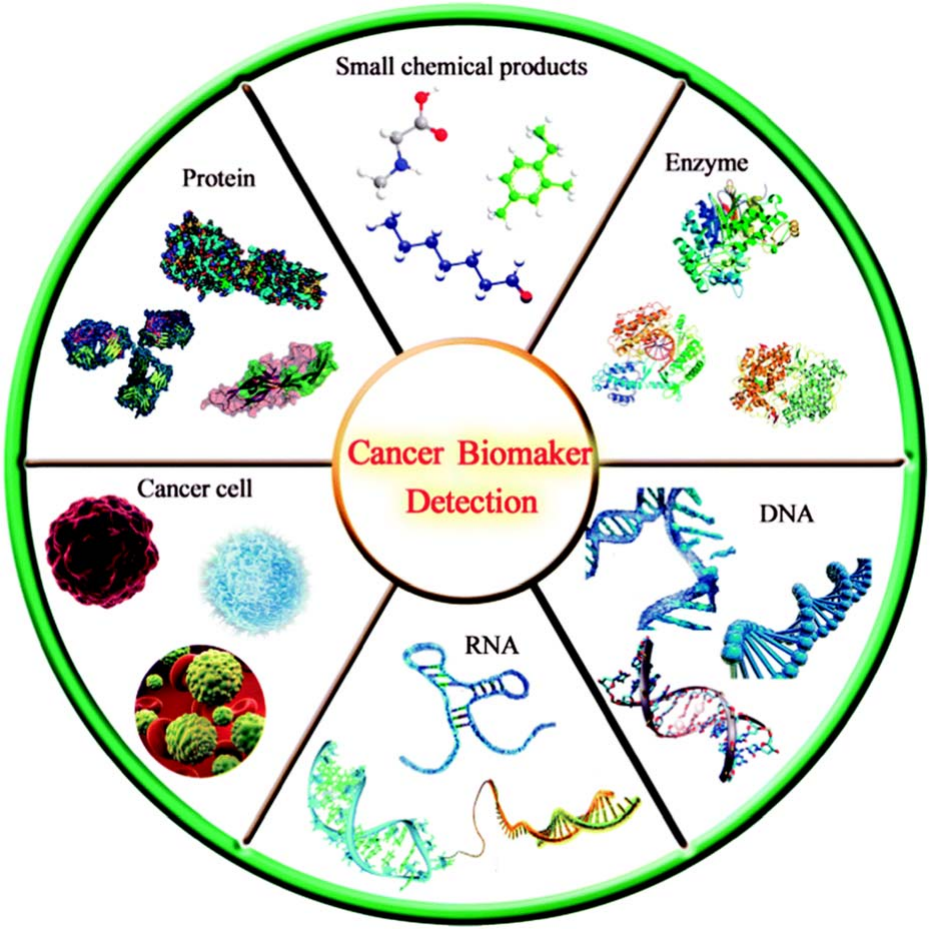
Schematic diagram of various tumor markers. Reproduced from ref. 71 with permission from Royal Society of Chemistry.

Tumor biomarkers are substances existing or produced by tumors or tumor hosts in response to the existence of tumors, including a variety of biomolecules such as proteins, DNA, mRNA, enzymes, metabolites, transcription factors and cell surface receptors (Fig. 10).^71,72^ Nowadays, tumor biomarkers have become a reliable tool for predicting the behavior of a variety of tumors, helping clinicians to determine the types of molecular disorders that lead to disease outbreaks. Tumor biomarkers from serum protein are widely used in clinical diagnosis, including CEA, epidermal growth factor receptor (EGFR), AFP, carbohydrate antigen 125 (CA125), carbohydrate antigen 19-9 (CA19-9), PSA and so on.^73^ Through the real-time detection of these tumor biomarkers, the disease of patients can be monitored, prognosis and treatment. For instance, Zhang *et al.*,^74^ proposed a new immunosensor strategy for the detection of cancer-specific protein biomarkers by combining specific target recognition with homogeneous transcription amplification. S. Roy *et al.*,^75^ developed a non-invasive, liquid-biopsy based assay by using circular RNAs (circRNAs) as a molecular biomarkers for early detection of gastric cancers. Xu *et al.*,^76^ developed a novel strategy for *in situ* isolating and directly detecting circulating tumor cell (CTC) from peripheral blood at single-cell resolution using black TiO_2_ (B-TiO_2_)-based Surface-Enhanced Raman Scattering (SERS) bio-probe on a microfilter.

At present, a variety of methods have been developed for sensitive detection of biomarkers. Because of the advantages of real-time measurement, low cost, high selectivity and sensitivity, immunosensor is very attractive in the detection of tumor markers.^77^ ELISA is commonly used for quantitative analysis of tumor biomarkers. However, this method requires professional and technical knowledge, complex procedures with high cost and low sensitivity, which limits its application in early detection and continuous monitoring.^78^ Due to the advantages of simple operation, fast response and high stability in practical detection, fluorescence immunosensor analysis is one of the most promising immunoassay methods at present. The introduction of functional nanomaterials has opened up a new way for the construction of fluorescence immunosensor.

### Detection of tumor biomarkers by fluorescence immunosensor constructed by functional nanomaterials

**Fig. 11:**
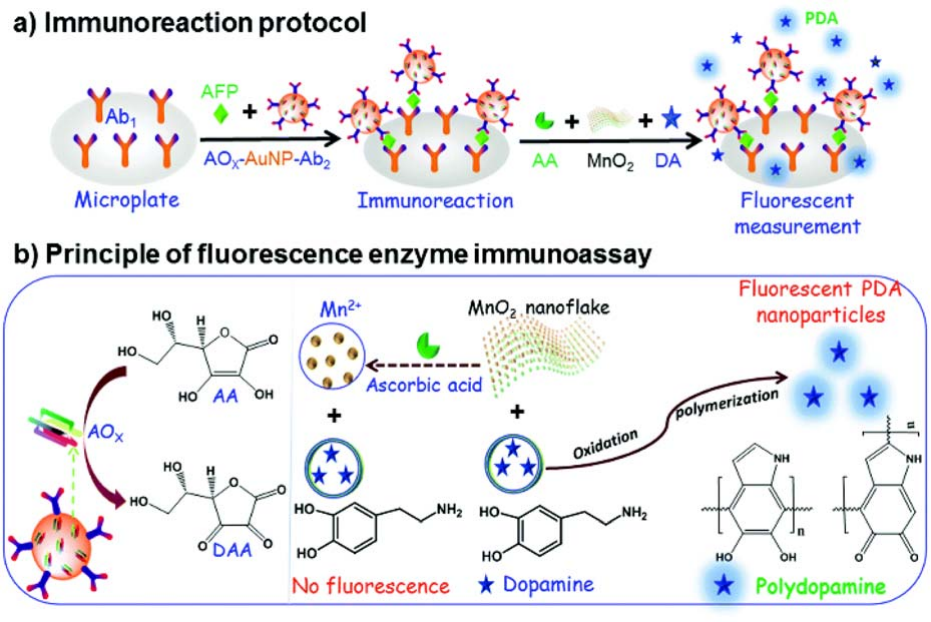
The fluorescence linked immunosorbent assay using AOx-AUNP-Ab_2_ as beacon antibody for the determination of AFP: (a) the immunoreaction protocol, (b) principle of fluorescence enzyme immunoassay. Reproduced from ref. 81 with permission from Royal Society of Chemistry.

AuNPs has been widely used in the field of biology and medicine because of its unique optical properties, high specific surface area and easy surface functionalization.^79^ The surface of AuNPs has a high affinity for mercaptan, disulfide, dithiocarbamate and amine, and is easy to couple with various biomolecules to form functional AuNPs for the construction of immunosensors.^80^ As shown in Fig. 11a, Lin *et al.*,^81^ designed a fluorescent enzyme-linked immunosorbent assay (FLISA) for the detection of AFP by using ascorbate oxidase (AOx) and secondary antibody labeled gold nanoparticles (AOx-AuNP-Ab_2_) as beacon antibodies. As shown in Fig. 11b, when there was no target, ascorbic acid (AA) decomposed manganese dioxide nanoparticles (MnO_2_) to form bivalent manganese ions, preventing dopamine (DA) from forming polydopamine (PDA). In the presence of the target AFP, AOx-AuNP-Ab_2_ was fixed to the microplate by double antibody sandwich, and the AOx on the sandwich immune complex could oxidize AA to form dehydroascorbic acid (DAA), which inhibited the decomposition of MnO_2_ nanoparticles, thus promoting the oxidation of DA by MnO_2_ nanoparticles and forming more PDA fluorescent nanoparticles. Therefore, a “signal on” fluorescent immunosensor system was constructed. A large amount of AOx modified onto AuNPs could promote the enzyme cascade amplification of the immune system and improved the sensitivity of the fluorescent immunosensor.

**Fig. 12:**
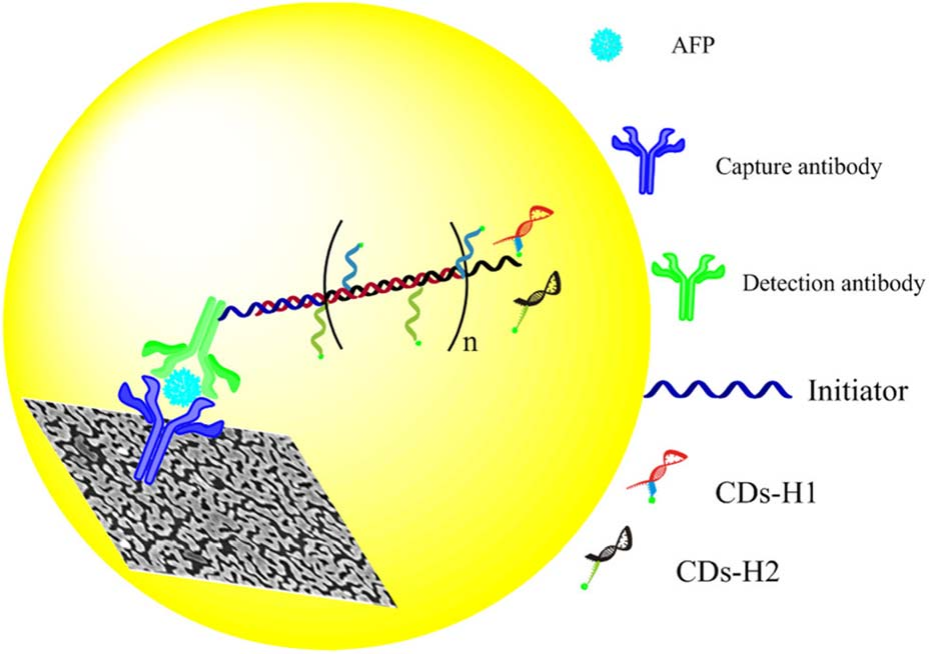
Schematic diagram of a fluorescence sensor based on HCR and MEF double amplification for sensitive detection of AFP. Reproduced from ref. 85 with permission from American Chemical Society.

Due to the surface plasmon resonance (SPR) effect of noble metal nanoparticles, it can affect the luminescence properties of fluorescent molecules, and enhance or quench the fluorescence of fluorescent molecules.^82^ When the fluorescent molecules are close to the surface of noble metal nanoparticles, the emission intensity of fluorescent molecules is greatly enhanced, which is called metal enhanced fluorescence (MEF).^83,84^ Due to the enhanced fluorescence of fluorescent molecules, the MEF effect can effectively improve the sensitivity of the immunosensor. Xu *et al.*,^85^ constructed a fluorescence sensor based on hybridization chain reaction (HCR) and MEF for sensitive detection of AFP (Fig. 12). The glass slides were covered with a plasmonic gold films conjugated with capture antibodies, and the detection antibodies conjugated with an oligonucleotide initiator were used to bind to AFP captured on the slide. Then, carbon quantum dots (CDs) labeled DNA hairpins (H1 and H2) were introduced to produce HCR reaction, resulting in the formation of copolymers containing a large number of CDs on the gold film surface. The SPR effect between CDs and gold film greatly increased the radiative decay rate and quantum yield of CDs, thus enhancing the fluorescence of CDs, while HCR achieved the secondary amplification of CDs fluorescence intensity. The dual-signal amplified fluorescence sensor had high sensitivity and wide linear range.

**Fig. 13:**
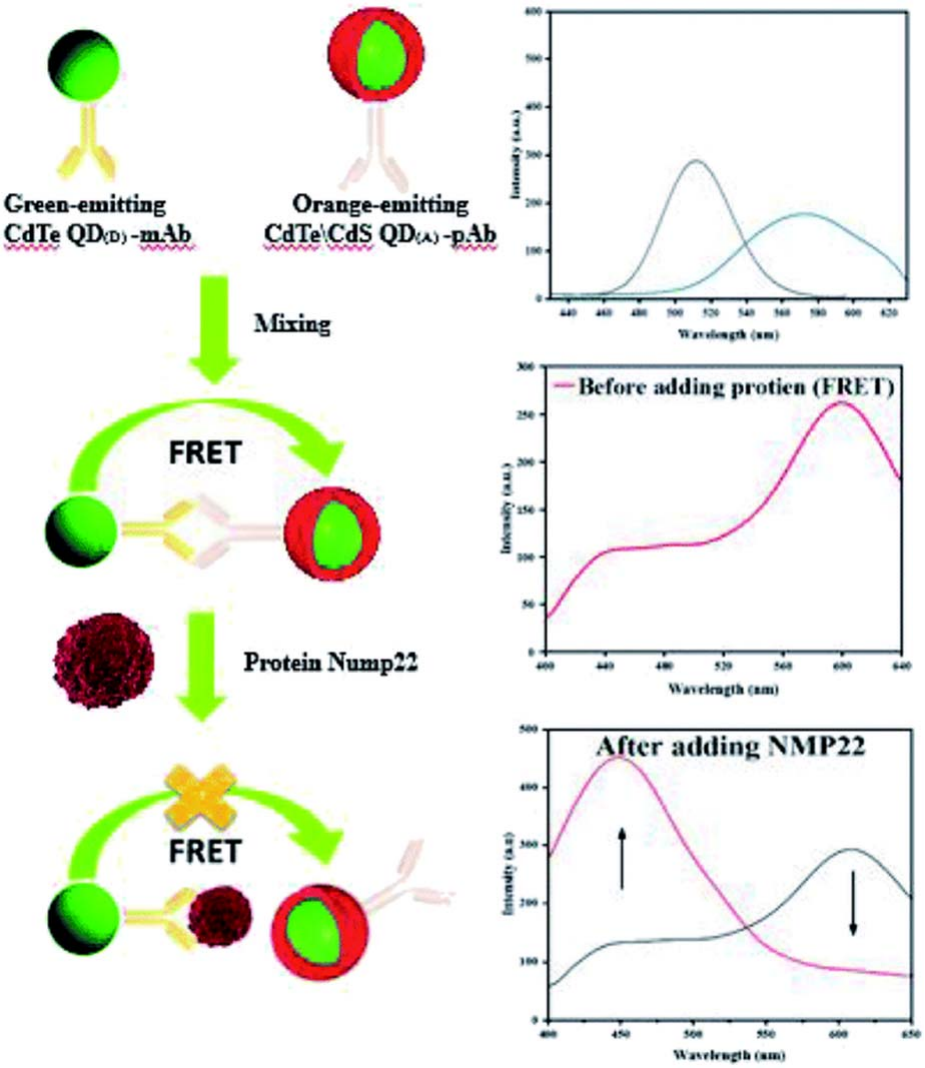
Schematic diagram of NMP22 detection using fluorescence immunosensing platform based on two different semiconductor quantum dots. Reproduced from ref. 87 with permission from Royal Society of Chemistry.

In the past two decades, quantum dots have become a hot spot in the development of new fluorescent nanomaterials.^8^ Quantum dots have strong fluorescence, good photostability and biocompatibility, and can be used as probes for the detection of biological analytes.^81^ In many studies, it has been found that most of the synthesized quantum dots are hydrophobic, so some precursors and ligands are commonly used to increase the surface hydrophilicity and promote their coupling with biomolecules.^86^ Semiconductor quantum dot is a popular fluorescent probe, and its application in fluorescence immunoassay has been studied widely. A fluorescence immunosensor platform based on two different semiconductor quantum dots has been reported for the sensitive detection of nuclear matrix protein 22.^87^ Cadmium telluride quantum dots (CdTeQD) were synthesized by using thioglycolic acid (TGA) as capping agent. CdTeQD were used to couple NMP22 monoclonal antibodies (mAb), and shell core quantum dots (CdTe/CdS) were synthesized from the solution of TGA and thiourea as shell precursors for coupling NMP22 polyclonal antibodies (pAb). As shown in Fig. 13, in the absence of NMP22, the two QDs of the coupling antibody formed an immune complex. Due to the close distance, they underwent FRET, resulting in the decrease of CdTeQD fluorescence and the enhancement of CdTe/CdS fluorescence. In the presence of NMP22, pAb was competitively replaced by NMP22, blocking FRET, enhancing CdTeQD fluorescence and decreasing CdTe/CdS fluorescence. The quantum dots used in this method are simple and low cost, and the fluorescence immunosensor is simple and sensitive.

The fluorescence measurement of a single emission peak is easily affected by many factors, which in turn affects the accuracy of the detection results. To improve the accuracy of detection results, ratiometric fluorescence method is applied for more and more biomolecule detection. Liu *et al.*,^88^ designed a ratiometric fluorescence immunoassay for the detection of AFP by using MnO_2_ nanoparticles, *o*-phenylenediamine (OPD) and fluorescent carbon nano-dots (FCNs). Primary antibody pre-adsorbed onto the well surface of a 96-well plate captured alpha-fetoprotein (AFP) and formed a sandwich complex with the secondary antibody coated on the MnO_2_ nanosheets. Then, FCNs and OPD were added into the wells, and MnO_2_ oxidized OPD to 2,3-diaminophenazine (DAP). FRET occurred between DAP and FCNs, DAP showed a strong fluorescence emission peak at 580 nm, while FCNs' fluorescence emission intensity decreased at 490 nm. The AFP was quantified by fluorescence intensity ratio between *I*_580_ and *I*_490_. The analytical performance of this ratiometric fluorescence immunoassay is obviously superior to the traditional single wavelength method. Although quantum dots have high fluorescence efficiency and photostability, they are easy to agglomerate as metal nanoclusters, which leads to the decrease of fluorescence intensity. To solve this problem, Li *et al.*,^89^ designed CdTe quantum dots (CdTe QDs) and gold nanoclusters (Au NCs) coated-silica nanospheres as labels. After sandwich immune reaction with immunomagnetic beads, carcinoembryonic antigen (CEA) was detected by fluorescence method.

Because aptamers have the characteristics of easy production, low cost,, easy labeling, long-term storage and high specificity, the application of aptamers in immunosensors has received widespread attention.^90^ S. Mohammadi *et al.*,^91^ reported a FRET-immunoassay based on the biospecific interactions between antibody and the corresponding antibody and aptamer for sensitive detection of breast cancer antigen (CA15-3). CA15-3 antibody-functionalized carbon dots were used as fluorescence donor and AuNPs labeled PAMAM-dendrimer/aptamer were used as quencher. The distance between CDs and AuNPs-PAMAM surface was shortened by sandwich immune reaction, so that the fluorescence of CDs was quenched to achieve the purpose of detection of CA15-3. The fluorescence immunoassay showed good stability and specificity in detection and analysis.

## Summary and outlook

In this paper, we summarize the progress in the detection of tumor biomarkers by using different immunosensors. The construction process of these immunosensors is not only relatively simple, without complex instruments, but also the amplified signal can be obtained by further modification. In addition, nanomaterials play an important role in the immunosensor, which makes the design of the immunosensor more flexible and diverse. These different immunosensors provide a new opportunity for the detection of tumor biomarkers. With the continuous development of nanozymes, high sensitive immunosensors can be designed by using the enzyme-like activity of nanomaterials.

At present, most immunoassays can only detect a single target in one test process. Therefore, it is necessary to develop a rapid and sensitive multiple analysis method to realize the quantitative detection of multiple targets at the same time.^92^ In the future research, it is of great significance to design and manufacture a portable, fast response, sensitive and point-of-care detection immunosensor,^93^ which can be achieved by using the bio-barcode assay technology using oligonucleotides.^94^ The development of promising fluorescent labels are very important for fluorescent immunosensors, such as semiconductor nanocrystals, metal nanomaterials, carbon materials, upconversion nanoparticles and rare earth materials, especially semiconductor QDs. Its high brightness and quantum yields, broad excitation spectrum and photochemical stability have become a novel generated fluorescent lable.^7^

Although a variety of fluorescent immunosensors have been constructed for the detection of tumor biomarkers, the sensors with simple construction, low cost, high sensitivity and good stability are still the focus of the current research.

## Author contributions

Juanjuan Huang: conceptualization, methodology, investigation, formal analysis, writing-original draft, writing-review & editing. Fenghuang Wei: conceptualization, methodology, investigation, formal analysis, writing – original draft, writing-review & editing. Yuling Cui: conceptualization, methodology, supervision. Li Hou: conceptualization, formal analysis, writing-review & editing, supervision, funding acquisition. Tianran Lin: conceptualization, formal analysis, writing-review & editing, supervision, funding acquisition.

## Conflicts of interest

There are no conflicts of interest to declare.

## Supplementary Material
